# Going beyond genetics to discover cancer targets

**DOI:** 10.1186/s13059-017-1238-7

**Published:** 2017-05-22

**Authors:** Gabriel J. Sandoval, William C. Hahn

**Affiliations:** 1 Dana-Farber Cancer Institute and Harvard Medical School, Brookline Ave, Boston, MA 02215 USA; 2 Broad Institute of Harvard and MIT, Main Street, Cambridge, MA 02142 USA

## Abstract

Two recent studies demonstrate the power of integrating tumor genotype information with epigenetic and proteomic studies to discover potential therapeutic targets in breast cancer.

## Limitations of genome sequencing data

The application of massively parallel sequencing technologies to characterize cancer genomes provides a foundation that not only has enabled the discovery of targets in particular cancers but also, in some cases, has informed the selection of therapeutic agents [1]. Nevertheless, it is clear that this information alone does not suffice to provide a complete picture of each patient’s tumor. For example, sequencing individual tumors usually yields a long list of mutated, amplified, or deleted alleles, for the majority of which we lack any functional information. Moreover, there are patients who harbor a mutant allele but do not respond to treatments targeting that allele. Furthermore, single-agent targeted therapies sometimes induce early responses but resistance inevitably occurs. Together, these observations indicate that additional information is needed to provide an understanding of cancer vulnerabilities and to operationalize precision medicine in cancer. Two recent breast cancer studies utilizing both a broad [2] and a focused approach [3] to interrogate protein and epigenetic modifications have suggested the types of additional information needed to allow us to understand cancer dependencies more fully.

## Breast cancer and phosphatidyl inositol 3-kinase

Breast cancer comprises of a complex and heterogeneous set of cancers now divided into several major subtypes according to their molecular features [4]. Although much has been learned from the study of breast cancer cell lines, these established cultures do not fully recapitulate the spectrum of human breast cancers. For these reasons, many investigators have begun to create collections of patient-derived xenografts (PDXs), which are propagated in immunodeficient animals. Although it is assumed that these models better recapitulate many aspects of human breast cancers, this has not been examined rigorously. Huang et al. [2] hypothesized that the use of a combination of genomic and quantitative proteomic approaches to study 24 breast cancer PDXs would allow them to characterize such tumors more fully. Specifically, they subjected each of these PDXs to whole-exome and RNA sequencing as well as a quantitative proteomic method based on mass spectrometry. The proteomic method, called isobaric tags for relative and absolute quantification (iTRAQ), allowed them to quantify proteins and phosphorylation sites. These analyses showed that the PDXs recapitulated particular breast cancer subtypes, suggesting that these types of PDX models are useful models of patient tumors. When they examined the directed phosphoproteomic data, the authors confirmed that the HER2 and phosphatidyl inositol 3-kinase (PI3K) pathways were active in tumors that harbored somatic activating mutations in these pathways, but also found evidence that the HER2 and PI3K pathways were activated in a subset of tumors that lacked mutations in these pathways. Treatment of these PDXs with clinical-grade compounds that target these pathways decreased tumor growth. Although many more models need to be interrogated before making the case to test this therapeutic hypothesis in a clinical setting, these studies provide strong evidence that the analysis of signaling pathways complements genomic analysis of tumors.

By contrast, a recent study by Baselga and colleagues [3] examined breast cancers in which mutations in the PI3K pathway are present, but in which resistance to treatment is problematic. These investigators used epigenetic profiling to study the interaction between two signaling pathways commonly found in breast cancer. Earlier studies had found that activating *PIK3CA* mutations occur frequently in estrogen receptor (ER)-positive breast cancers [5], but treatment of such *PIK3CA-*mutant cancers with potent PI3K inhibitors induces an increase in ER-driven transcriptional programs that contribute to clinical resistance [6]. To investigate how this resistance arises, Baselga and colleagues [3] interrogated the state of chromatin at the ER locus in cells with and without the PI3Kα inhibitor BYL719. They found that BYL719 treatment induced an open chromatin state, allowing the ER and the pioneering factor FOXA1 to upregulate target genes. This ER-dependent gene activation was regulated by the H3K4 methyltransferase KMT2D, which itself is inactivated by phosphorylation by AKT1, resulting in a closed chromatin state. This study suggests that KMT2D is an attractive therapeutic target for ER-positive breast cancer patients treated with PI3K inhibition. More generally, it highlights the importance of deciphering mechanisms, such as the mutation of *PIK3CA*, that modulate the activity of oncogenic events.

## The bigger picture

Although the genomic characterization of tumors identifies some of the mechanisms that drive cancer initiation and progression, these two studies demonstrate that other, non-genetic mechanisms may also activate key signaling pathways in cancers. Like the cancer-associated changes in signaling pathways revealed in these studies, disruption of the normal mechanisms that regulate the post-translational modification of proteins contributes directly to a number of cancer phenotypes. Indeed, The Cancer Genome Atlas (TCGA) includes antibody-based assessment of protein phosphorylation for many studies. Furthermore, the National Cancer Institute Clinical Proteomic Tumor Analysis Consortium (CPTAC) recently performed proteogenomic analysis on TCGA breast cancer samples to determine whether novel therapeutic opportunities could be discovered by connecting the genome to the proteome [7]. This analysis revealed important insights into the consequences of somatic mutations and led to the identification of potential druggable kinases that would have gone undetected by genomic analysis alone. In addition to proteomics, the development of methods to interrogate several epigenetic marks has facilitated the profiling of epigenetic states in both normal and diseased tissue. Similar to the comprehensive proteome analysis performed by TCGA, the NIH Roadmap Epigenomics Consortium has begun to generate human epigenome data to further reveal how epigenetic alterations contribute to disease [8]. Although it is clear that further work is necessary to demonstrate that the altered signaling and epigenetic profiles identify tumors that will respond to therapeutic interventions targeting the altered signaling pathways, these studies provide a rationale for a way to discover this information in patient tumors.

## Barriers to implementation

At present, a number of challenges limit the potential to obtain information about potential druggable targets in cancer by proteomic methods. Although recent advances in proteomic technologies allow the large-scale identification of protein modifications in both living tissue and stored tumor tissue samples, performing such analyses in the clinical setting will be difficult. For example, current proteomic approaches are often limited by the quality of the protein samples screened, which makes obtaining quality data from a limited set of patient samples difficult. Although new mass spectrometers will increasingly permit the unbiased interrogation of proteins in complex mixtures, antibodies still represent an important tool for purifying and investigating protein complexes and their interacting partners. This limits analyses to specific antibody epitopes and necessitates strong interactions that withstand purification steps. Moreover, the handling of samples that are destined for proteomic and epigenetic analyses is much more dependent on conditions than is the isolation of nucleic acids. Indeed, variations in how samples are collected, including the time and even the location, have been shown to create artifactual differences among samples [9, 10]. Rigorous protocols are therefore required to ensure that samples are handled in ways that allow for unbiased analyses from many sources.

A key challenge ahead will be to define the optimal information set that will complement genetic analyses of human tumors so as to allow the identification of cancers that are likely to respond to specific therapeutic agents. In addition to the methods described in the two reports highlighted here, the number and diversity of proteomic and epigenetic markers is large and growing. Further work will be required to define the markers that are informative and to optimize methods that can detect these markers in clinical settings. Nevertheless, these studies highlight the value of overcoming these challenges so that different types of tumor data can be integrated to identify targets and biomarkers that have the potential to expand the power and implementation of precision medicine.

## Abbreviations

ER: Estrogen receptor
PDX: Patient-derived xenograft
PI3K: Phosphatidyl inositol 3-kinase
TCGA: The Cancer Genome Atlas
